# Seroprevalence of SARS-CoV-2 in a Cohort of Patients with Multiple Sclerosis under Disease-Modifying Therapies

**DOI:** 10.3390/jcm11092509

**Published:** 2022-04-29

**Authors:** Agustín Sancho-Saldaña, Anna Gil Sánchez, Bibiana Quirant-Sánchez, Lara Nogueras, Silvia Peralta, Maria José Solana, Cristina González-Mingot, Yhovanni Gallego, Laura Quibus, Cristina Ramo-Tello, Silvia Presas-Rodríguez, Eva Martínez-Cáceres, Pascual Torres, José Vicente Hervás, Joan Valls, Luis Brieva

**Affiliations:** 1 Neurology Department, Hospital Universitario Arnau de Vilanova, IRB Lleida, 25198 Lleida, Spain; agustinsanchosaldana@gmail.com (A.S.-S.); solanamoga@gmail.com (M.J.S.); crismingot@hotmail.com (C.G.-M.); yoga253@hotmail.com (Y.G.); lquibusr@gmail.com (L.Q.); 2 Neuroimmunology Group, Institut de Recerca Biomèdica, Universitat de Lleida, 25001 Lleida, Spain; agil@irblleida.cat (A.G.S.); lara.noguerasp@gmail.com (L.N.); pascual.torres@udl.cat (P.T.); 3 Immunology Division, Hospital Germans Trias i Pujol, LCMN, 08916 Badalona, Spain; bquirant.germanstrias@gcat.cat (B.Q.-S.); emmartinez.germanstrias@gencat.cat (E.M.-C.); 4 Department of Cell Biology, Physiology, Immunology, Autonomous University, Bellaterra, 08193 Barcelona, Spain; 5 Multiple Sclerosis Foundation from Lleida, 25198 Lleida, Spain; sipem@hotmail.com; 6 Multiple Sclerosis and Clinical Neuroimmunology Unit, Neurosciences Department, Hospital Germans Trias i Pujol, 08916 Badalona, Spain; cramot@gmail.com (C.R.-T.); spresas.germanstrias@gencat.cat (S.P.-R.); 7 Hospital de Sant Joan Despí Moisès Broggi, 08970 Sant Joan Despí, Spain; josevicente.hervas.garcia@gmail.com; 8 Biostatistics Group, Institut de Recerca Biomèdica de Lleida, 25198 Lleida, Spain; joanvallsmarsal@gmail.com

**Keywords:** multiple sclerosis, COVID-19, SARS-CoV-2, DMT, seroprevalence

## Abstract

Background: Disease-modifying therapies (DMTs) used to treat multiple sclerosis (MS) alter the immune system and therefore increase the risk of infection. There is growing concern about the impact of COVID-19 on patients with MS (pwMS), especially those treated with DMTs. Methods: This is a single-center prospective observational study based on data from the Esclerosis Múltiple y COVID-19 (EMCOVID-19) study. Demographic characteristics, MS history, laboratory data and SARS-CoV-2 serology, and symptoms of COVID-19 in pwMS treated with any DTM were extracted. The relationship among demographics, MS status, DMT, and COVID-19 was evaluated. Results: A total of 259 pwMS were included. The administration of interferon was significantly associated with the presence of SARS-CoV-2 antibodies (26.4% vs. 10.7%, *p* = 0.006). Although patients taking interferon were significantly older (49.1 vs. 43.5, *p* = 0.003), the association of interferon with the presence of SARS-CoV-2 antibodies was still significant in the multivariate analysis (*OR* 2.99 (1.38; 6.36), *p* = 0.006). Conclusions: According to our data, pwMS present a higher risk of COVID-19 infection compared with results obtained from the general population. There is no evidence of a worse COVID-19 outcome in pwMS. DMTs did not significantly change the frequency of COVID-19, except for interferon; however, these findings must be interpreted with caution given the small sample of pwMS taking each DMT.

## 1. Introduction

Since its origin in Wuhan, China in December 2019, severe acute respiratory syndrome coronavirus 2 (SARS-CoV-2) causing Coronavirus disease 2019 (COVID-19) has rapidly become pandemic [1], affecting countries worldwide. COVID-19 has a wide range of clinical manifestations, ranging from no symptoms to life-threatening acute respiratory distress [2]. 

Spain is among the countries more heavily affected by COVID-19. Lleida is a region in the northwest of Spain, in Catalonia, with a reported seroprevalence ranging from 3.8% [3] (July 2020) to 10.4% (November 2020) and with a cumulated seroprevalence of 12.2%, according to the ENE-COVID study carried out by the Spanish Ministry of Health to determine the seroprevalence of SARS-CoV-2 in the Spanish population [4]. 

Multiple sclerosis (MS) is a chronic, inflammatory, and demyelinating disease in which the autoimmune system attacks the myelin sheath in the central nervous system [5]. It is a leading cause of neurologic symptoms in young adults and has no known cure.

The disease-modifying therapies (DMTs) used to treat MS alter the immune system, thus increasing the risk of infection, mainly in the upper respiratory and urinary tracts [6]. There is growing concern about the impact of COVID-19 on patients with multiple sclerosis (pwMS). Currently, there is no evidence that pwMS taking DMTs are more susceptible to developing COVID-19 or more likely to present a worse outcome. Data from an Italian registry suggest that COVID-19 is more severe in patients with progressive MS, over 50 years of age, or with a higher Expanded Disability Status Scale (EDSS) score. The infection fatality risk for SARS-CoV-2 in MS was 1.66%. Most nonsurvivors were not taking DMTs [7], and it has even been suggested that immunosuppression in pwMS taking certain DMTs may protect against severe COVID-19 infection [8]

Some studies [9,10,11,12] have suggested that each of the DMTs used in MS has a different impact on COVID-19 infection (no risk: interferon beta and glatiramer; low risk: teriflunomide, dimethyl fumarate, and natalizumab; intermediate or high risk: fingolimod, anti-CD20 therapies, cladribine, and alemtuzumab).

There are sparse data on the complex natural immunity to SARS-CoV-2 at the population level. A general population study in Catalonia in which a well-validated multiplex serology test was performed in around 5000 subjects revealed a seroprevalence of 18.1% in adults, and extrapolation of the results to the general population of Catalonia suggested a seroprevalence of 15.3% [13]. Antibodies persisted up to 9 months after infection. Immune profiling of infected individuals revealed that the more severe the infection, the more robust the seroresponse, with a shift towards IgG over IgA and antispike over antinucleocapsid responses. Asymptomatic COVID-19 infections account for 28.7% [14], and these patients are more likely to show greater IgA than IgG responses compared to those with more severe disease.

In this study performed in the province of Lleida, Spain, we evaluated the prevalence and severity of SARS-CoV-2 in pwMS taking DMTs and its relationship with each DMT. We hypothesize that pwMS taking DMTs are more susceptible to SARS-CoV-2 infection.

## 2. Material and Methods

### 2.1. Study Design and Patients

This is a single-center, prospective, observational study based on data from the prospective ongoing Esclerosis Múltiple y COVID-19 (EMCOVID-19, by its Spanish acronym) study carried out by 20 centers in Spain that aims to evaluate the seroprevalence of SARS-CoV-2 in a large cohort of pwMS treated with DMTs in order to evaluate the correlation between MS and COVID-19.

In EMCOVID-19, patients attended two visits (baseline and 6 months) in which they were asked about their latest or recent manifestations of COVID-19 and their MS symptoms, and a blood sample was taken.

All patients diagnosed with MS treated with any DMT in the MS unit in the Hospital Universitario Arnau de Vilanova, Lleida, Spain study were included. Data from the baseline EMCOVID-19 visit were extracted. Baseline characteristics (sex, age, pregnant/not pregnant, smoker history, MS type, and EDSS), MS history (time from MS diagnosis, time from first symptoms, time from latest relapse, use of glucocorticoids in the previous 3 months, and current DMT), laboratory data (lymphocyte count), and symptoms of COVID-19 were recorded, and the correlation among any of these characteristics and the presence of antibodies for SARS-CoV-2 in serum was analyzed. Lymphopenia was defined as total lymphocytes <1000/μL

Patients with IgG, IgM, or IgA antibodies against SARS-CoV-2 were considered confirmed cases for SARS-CoV-2 infection and classified as symptomatic or asymptomatic.

The results of seroprevalence in this study were compared with those obtained in the general population. Epidemiological data of COVID-19 cases confirmed by serological analysis were obtained from ENE-COVID, a Spanish nationwide, population-based seroepidemiological study performed by the Ministry of Health, Consumer Affairs and Social Welfare [3,4].

### 2.2. Blood Samples

Peripheral blood samples were taken between March 2020 and September 2020, before the start of COVID-19 vaccination in Spain (28 December 2020). Samples were centrifuged and frozen at −80 °C.

ELISA was used to determine IgG, IgM, and IgA against SARS-CoV-2 using 3 recombinant antigens: nucleocapsid, S1, and S2 dominion (Diapro^®^, Sesto San Giovanni, Italy).

### 2.3. Statistical Analysis

Mean (and standard deviation) and absolute frequency (and percentage) were used to describe the variables analyzed, and the median and interquartile range was also reported when appropriate. Bivariate tests, such as the chi-square test, *t*-test, and Anova (when a parametric test was required) or Fisher’s and Kruskal–Wallis tests (when a nonparametric test was required) were performed to evaluate the correlation between variables. Prevalence was calculated as a percentage with a 95% confidence interval (CI). Simple logistic regression models were used to estimate odds ratios (OR) to assess the association between different risk factors and positive immunization status. A stepwise multiple logistic regression model was constructed to determine factors with a significant correlation. All analyses were performed using R software, setting the threshold for significance at 0.05.

## 3. Results

A total of 259 patients were included, with a median age of 44.3; 171 (66%) were female, and 88 (33%) were male; 58 patients (23.7%) were active smokers. In terms of MS, 223 (86.1%) presented relapsing-remitting multiple sclerosis (RRMS); 21 (8.11%) presented secondary progressive multiple sclerosis (SPMS), and 15 patients (5.79%) presented primary progressive multiple sclerosis (PPMS). One hundred sixty-seven patients (66.3%) had not had a relapse in the previous year, and only 13 patients (5.2%) had received glucocorticoids in the previous 3 months to treat a relapse.

One hundred thirty-three patients (51.3%) were taking some kind of platform DMT (immunomodulatory treatment), and 126 (49.1%) were taking a high-activity DMT (immunosuppressive treatment). More information about baseline characteristics and treatments is shown in Table 1. 

One hundred thirty-five patients (52.1%) had lymphopenia (<1000 lymphocytes) of which 22 (16.3%) had severe lymphopenia (grade 4; <200 lymphocytes).

Fifty-three (20.46%) patients were positive for IgG, IgM, or IgA antibodies against SARS-CoV-2: 28 (10.9%) were IgG positive; 29 (11.4%) were IgM positive, and 17 (6.75%) were IgA positive.

In total, 14 patients (5.43%) had COVID-19 symptoms. Half of these patients (7/14) had a fever and/or cough; 4 patients (28%) had nasal congestion and/or dysphonia, and 3 (21%) patients had mild or moderate dyspnea. Fatigue and/or headache was found in 3 patients (21%), and 1 patient had anosmia (7.1%). One patient received empirical treatment with azithromycin, and only 1 patient required hospitalization. This patient received ocrelizumab and presented with fever, moderate dyspnea, and bilateral pneumonia. He received hydroxychloroquine and oxygen therapy and made a good recovery after 15 days of hospitalization.

Among symptomatic patients, three (21%) were taking glatiramer, two patients dimethyl fumarate (14.2%), two patients teriflunomide, two patients ocrelizumab, one patient interferon (7.1%), one patient cladribine, one patient natalizumab, and one patient alemtuzumab.

The binary analysis showing differences between seropositive and seronegative patients is shown in Table 2.

Interferon was significantly associated with the presence of SARS-CoV-2 antibodies (26.4% vs. 10.7%, *p =* 0.006). Although patients on interferon were significantly older (49.1 vs. 43.5, *p =* 0.003), the association between interferon and SARS-CoV-2 antibodies was still significant in the multivariate analysis (*OR* 2.99 (1.38; 6.36), *p =* 0.006). Alemtuzumab was also associated with the presence of SARS-CoV-2 antibodies (7.7% vs. 4.37%, *p =* 0.31), but this was not statistically significant. No association was found with the remaining DMTs (Table 2).

## 4. Discussion

It is still unclear whether pwMS have an increased susceptibility to COVID-19 and worse outcomes compared with the general population. Describing the characteristics of the immune response in specific autoimmune pathologies, such as MS, that are treated with immune system-modifying drugs can help us understand how SARS-CoV-2 affects this population and how we can minimize the risks.

In a previous study [15], 18 out of 76 pwMS (23.7%) were hospitalized; 8 (10.5%) had COVID-19 critical illness or related death. A similar proportion was reported in other studies [16]. Factors associated with worse outcomes were similar to those found in the general population (older age, presence of comorbidities, progressive disease, and nonambulatory status), and DMT use was not associated with a worse prognosis [15]. Although the proportion of hospitalized patients in the latter study is considerably higher than that reported here, all their pwMS had symptoms suggestive of COVID-19, which constitutes a selection bias. In a survey study performed in Barcelona, a higher incidence of COVID-19 was found in pwMS compared to the general population (COVID-19 was confirmed in 5 patients (1.2%) by PCR and suspected in 46 (11.3%)) [16]. In this study, only symptomatic patients or those admitted to hospital underwent PCR testing, which could explain the lower frequency of COVID-19 cases compared to our data. In this sample, the prevalence of COVID-19 among pwMS treated with DMT is notably higher than that reported in a previous study performed in Lleida (20.4% vs. 12.2%) [3].

In our cohort, 94.6% of patients were asymptomatic. Symptomatic patients presented with mild symptoms, and hospitalization was only required in one case treated with ocrelizumab. Symptoms, however, were not associated with lymphopenia or any specific DMT.

Immune response to SARS-CoV-2 plays a critical role in the development of acute respiratory distress syndrome (ARDS) and determines prognosis due to the exacerbation of inflammatory components after dysregulation of the immune system [17]. Based on the hypothesis that an overactive immune response could cause clinical deterioration in SARS-CoV-2 infection, it has been suggested that immunosuppressive or immunomodulatory therapies could protect against some COVID-19 complications [18,19].

Our results show that treatment with a specific DMT was not significantly associated with higher seroprevalence, except in patients taking interferon, and the risk of infection was not higher in patients taking immunosuppressive drugs vs. those taking immunomodulatory drugs.

Interferons are naturally occurring cytokines that participate in a wide range of anti-inflammatory processes [20]. Due to its putative antiviral effect, it seems unlikely that interferon would increase susceptibility to infection or would negatively influence the immune response against SARS-CoV-2 [21]. We think that the higher seroprevalence among patients taking interferon could be explained by interferon having less effect on the immune system resulting in a more appropriate humoral response. A meta-analysis of clinical trials revealed that early administration of interferon-β in combination with antiviral drugs was a promising therapeutic strategy against COVID-19 [22]; however, this was not confirmed in a recent clinical trial [23]. Another DMT, fingolimod, is thought to be potentially useful to treat COVID-19 once pneumonia is established, due to some type of ‘polycytokine’ inhibiting properties that may have more beneficial effects compared to selective cytokine inhibitors [24]. The risk of severe COVID-19 in pwMS taking fingolimod or siponimod appears to be similar to the general population [25]. All these findings could support the use of immunosuppressants to reduce the cytokine storm caused by COVID-19, and therefore prevent ARDS [26,27,28].

Based on this new evidence, pwMS treated with DMTs could be more susceptible to SARS-CoV-2 infection for various reasons. For example, immunosuppression derived from some DMTs could make pwMS more susceptible to COVID-19 infection (higher percentage of infections by SARS-CoV-2) without affecting the capacity of the immune response to fight the virus (most infected patients were asymptomatic). However, we found no significant association between lymphopenia and susceptibility to SARS-CoV-2 infection. A recent study characterizing humoral immunity in mRNA-COVID-19 MS vaccinees treated with high-efficacy DMTs found that some developed a humoral response despite a normal absolute lymphocyte count [29]. The entire sample of patients under treatment with DMT in our center underwent ELISA, and all reported cases were confirmed and retested, allowing us to detect asymptomatic cases. In a previously published study performed in Barcelona, DMT was not associated with a risk of infection [16]. In a cross-sectional study performed in Italy between 11 May and 15 June 2020, the prevalence of SARS-CoV-2 IgG/IgM in pwMS, including those receiving systemic immunosuppression treatment, was low (2.9%) and similar to the general population [30]. However, this study used a less sensitive test (lateral flow), which could explain this discrepancy. Fewer patients on ocrelizumab had antibodies against SARS-CoV-2, although this difference was not significant. Ocrelizumab is a humanized monoclonal antibody that targets CD20 on the surface of B-cells, causing prolonged selective B-cell depletion and depleting antibody production. The authors of a recent case series [31] concluded that B-cell depleting therapies, such as rituximab and ocrelizumab, might be associated with greater susceptibility to COVID-19. In this case, the diagnosis of COVID-19 was based on clinical and radiological findings, but not on a serologic test, such as that used in our study. In this regard, Zabalza et al. also reported less serological response in patients on anti-CD20 therapies (15.8%) than those on other DMTs (48.8%; *p* = 0.045) or no DMTs (68.4%; *p* = 0.003) [32]. Some authors suggest pwMS taking B-cell-depleting therapies could have a worse COVID-19 prognosis [11,33]. However, others suggest that anti-CD20 does not appear to contribute to the risk of infection by SARS-CoV2 [34]. We believe that patients on B-cell-depleting therapies may be more prone to COVID-19 infection because they produce low, short-lived antibody titers. Although infection is more likely to be due to T-cell dysfunction, B-cells play an important role in T-cell regulation [35].

This study has some strengths and limitations. Among its strengths, pwMS in Lleida were managed in a single MS unit that is far from other MS units in Catalonia. Therefore, the sample is representative of the MS population in the province of Lleida. In addition, the ELISA test used in the analysis is more sensitive to antibodies against SARS-CoV-2 than the techniques used in other studies. This allowed us to detect most of COVID-19 cases, regardless of time of infection or severity. Another strength of this study is the availability of a reference population in the same time period and epidemiological context from the ENECOVID study.

In terms of limitations, this is a single-center study that used a relatively small sample of patients taking any DMTs to assess their relationship with immune serologic status. Cell-mediated immunity against SARS-CoV-2 was not evaluated, and the ELISA test could mask false positives with other coronaviruses. Finally, although we used data from the ENECOVID study as our reference population, we did not have a true control group to compare our findings.

In conclusion, according to the collected data, pwMS (especially those with RRMS) had a higher seroprevalence of COVID-19 in comparison with previous reports obtained by serological analysis of the general population, although most of them were asymptomatic. There is no evidence of a worse COVID-19 outcome in patients affected by MS. DMTs did not significantly change the severity of COVID-19; however, these findings must be interpreted with caution given the small number of pwMS taking each DMT. Seroprevalence was higher in patients taking interferon, but this could be explained by a “healthier” humoral response against COVD-19 instead of an increased susceptibility to infection. Immunosuppressive drugs did not increase the risk of infection compared with immunomodulatory drugs. This, in turn, may raise other questions regarding the effect of ongoing vaccines in pwMS, especially in those who have already had COVID-19.

To the best of our knowledge, this is the largest prospective study analyzing the seroprevalence of SARS-CoV-2 in pwMS and its relationship with DMTs in Spain; however, multicenter studies with even larger sample are warranted to add clarity to some of the questions that concern both neurologists and patients.

## Figures and Tables

**Table 1:** Demographics, clinical characteristics, DMT, and COVID-19 immune status.

Baseline Characteristics	*N* = 259
Age, mean (*SD*)	44.3 (10.3)
Female sex, *n* (%)	171 (66.0)
Pregnant, *n* (%)	0 (0)
EDSS, mean (*SD*)	2.00 (2.19)
Current smoker, *n* (%)	58 (23.7)
Former smoker, *n* (%)	49 (20)
Never smoker, *n* (%)	138 (56.3)
Hypertension, *n* (%)	31 (19.6)
Diabetes, *n* (%)	8 (5.93)
Obesity, *n* (%)	24 (15.9)
MS type, *n* (%)	
RRMS	223 (86.1)
PPMS	15 (5.79)
SPMS	21 (8.1)
No relapse in previous year	221 (85.3)
Steroids in previous 3 months, *n* (%)	13 (5.2)
Platform DMT, *n* (%)	
Interferon	36 (13.9)
Glatiramer	15 (5.79)
Teriflunomide	33 (12.7)
Dimethyl Fumarate	49 (18.9)
Second-line DMT, *n* (%)	
Fingolimod	18 (6.95)
Natalizumab	24 (9.27)
Rituximab	13 (5.02)
Ocrelizumab	41 (15.8)
Cladribine	17 (6.56)
Alemtuzumab	13 (5.2)
Lymphopenia, *n* (%)	135 (52.1)
≤200 (Grade 4)	22 (16.3)
201–500 (Grade 3)	19 (14.1)
501–800 (Grade 2)	34 (25.2)
801–1000 (Grade 1)	60 (44.4)
Sign and symptoms of COVID19 *n* (%)	14 (5.43)

DMT: Disease-modifying treatment; EDSS: Expanded Disability Status Scale; MS: Multiple sclerosis; PPMS: progressive multiple sclerosis; RRMS: relapsing-remitting multiple sclerosis; SPMS: secondary progressive multiple sclerosis.

**Table 2:** Demographics, clinical characteristics, DMT, and COVID-19 immune status.

	MS Negative for SARS-CoV-2 IgG/IgM/IgA (*N* = 206)	MS Positive for SARS-CoV-2 IgG/IgM/IgA (*N* = 53)	*p*
Age, median (IQR)	44.0 (37.0–50.0)	47.0 (41.0–53.0)	0.076
Female sex, *n* (%)	133 (64.6)	38 (71.7)	0.41
EDSS, median (IQR)	1.50 (0.0–3.4)	1.00 (0.0–2.5)	0.053
Current smoker, *n* (%)	49 (25.3)	9 (17.6)	0.52
MS type, *n* (%)			0.269
RRMS	177(85.9)	46 (86.8)	
PPMS	14 (6.8)	1 (1.89)	
SPMS	15 (7.28)	6 (11.3)	
Steroids previous 3 months, *n* (%)	10 (5.08)	3 (5.66)	1
Hypertension, *n* (%)	23(18.7)	8(22.9)	0.76
Diabetes, *n* (%)	6 (5.6)	2 (6.9)	0.68
Obesity, *n* (%%)	18 (15.3)	6 (18.2)	0.89
Lymphopenia, *n* (%)	105 (51)	30 (56.6)	0.56
Platform DMT, *n* (%)	103 (50)	30 (56.6)	0.48
Interferon	22 (10.7)	14 (26.4)	0.006
Glatiramer	13 (6.31)	2 (3.77)	0.77
Teriflunomide	28 (13.6)	5 (9.43)	0.56
Dimethyl Fumarate	40 (19.4)	9 (17)	0.83
Second-line DMT, *n* (%)	103 (50)	23 (43.4)	0.48
Fingolimod	15 (7.28)	3 (5.66)	1
Natalizumab	20 (9.7)	4 (7.55)	0.79
Rituximab	11 (5.34)	2 (3.77)	1
Ocrelizumab	34 (16.5)	7 (13.2)	0.65
Cladribine	14 (6.8)	3 (5.6)	1
Alemtuzumab	9 (4.37)	4 (7.75)	0.31
Lymphopenia, *n* (%)	100 (48.5)	25 (47.2)	0.98

DMT: Disease-modifying treatment; EDSS: Expanded Disability Status Scale; IQR: interquartile range; MS: Multiple sclerosis.

## References

[1] Cucinotta D., Vanelli M. (2020). WHO declares COVID-19 a pandemic. Acta Biomed..

[2] Mao L., Wang M., Chen S., He Q., Chang J., Hong C., Zhou Y., Wang D., Miao X., Hu Y. (2020). Neurological Manifes-tations of Hospitalized Patients with COVID-19 in Wuhan, China: A Retrospective Case Series Study. SSRN Electron. J..

[3] Pollan M., Perez-Gomez B., Pastor-Barriuso R., Oteo J., Hernán M.A., Pérez-Olmeda M., Sanmartín J.L., Fernández-García A., Cruz I., Fernández de Larrea N. (2020). A Population-Based Seroepidemiological Study of SARS-CoV-2 in Spain (ENE-COVID). SSRN Electron. J..

[4] Estudio ENE-COVID Cuarta Ronda (2020). Estudio Nacional de Sero-Epidemiología de La Infección Por SARS-COV-2 En España 15 de Diciembre de 2020. https://www.sanidad.gob.es/gabinetePrensa/notaPrensa/pdf/15.12151220163348113.pdf.

[5] Reich D.S., Lucchinetti C.F., Calabresi P.A. (2018). Multiple Sclerosis. N. Engl. J. Med..

[6] Grebenciucova E., Pruitt A. (2017). Infections in Patients Receiving Multiple Sclerosis Disease-Modifying Therapies. Curr. Neurol. Neurosci. Rep..

[7] Sormani M.P. (2020). An Italian programme for COVID-19 infection in multiple sclerosis. Lancet Neurol..

[8] Giovannoni G., Hawkes C., Lechner-Scott J., Levy M., Waubant E., Gold J. (2020). The COVID-19 pandemic and the use of MS disease-modifying therapies. Mult. Scler. Relat. Disord..

[9] Reder A.T., Centonze D., Naylor M.L., Nagpal A., Rajbhandari R., Altincatal A., Kim M., Berdofe A., Radhakrishnan M., Jung E. (2021). COVID-19 in Patients with Multiple Sclerosis: Associations with Disease-Modifying Therapies. CNS Drugs.

[10] Hada M., Mosholder A.D., Leishear K., Perez-Vilar S. (2022). Systematic Review of Risk of SARS-CoV-2 Infection and Severity of COVID-19 with Therapies Approved to Treat Multiple Sclerosis. Neurol. Sci..

[11] Sormani M.P., De Rossi N., Schiavetti I., Carmisciano L., Cordioli C., Moiola L., Radaelli M., Immovilli P., Capobianco M., Trojano M. (2021). Disease-Modifying Therapies and Coronavirus Disease 2019 Severity in Multiple Sclerosis. Ann. Neurol..

[12] Simpson-Yap S., De Brouwer E., Kalincik T., Rijke N., Hillert J.A., Walton C., Edan G., Moreau Y., Spelman T., Geys L. (2021). Associations of Disease-Modifying Therapies With COVID-19 Severity in Multiple Sclerosis. Neurology.

[13] Karachaliou M., Moncunill G., Espinosa A., Castaño-Vinyals G., Jiménez A., Vidal M., Santano R., Barrios D., Puyol L., Carreras A. (2021). Infection induced SARS-CoV-2 seroprevalence and heterogeneity of antibody responses in a general population cohort study in Catalonia Spain. Sci. Rep..

[14] Pérez-Gómez B., Pastor-Barriuso R., Pérez-Olmeda M., Hernán A.M., Oteo-Iglesias J., de Larrea N.F., Fernández-García A., Martín M., Fernández-Navarro P., Cruz I. (2021). ENE-COVID nationwide serosurvey served to characterize asymptomatic infections and to develop a symptom-based risk score to predict COVID-19. J. Clin. Epidemiol..

[15] Parrotta E., Kister I., Charvet L., Sammarco C., Saha V., Charlson R.E., Howard J., Gutman J.M., Gottesman M., Abou-Fayssal N. (2020). COVID-19 outcomes in MS: Observational Study of Early Experience from NYU Multiple Sclerosis Comprehensive Care Center. Neurol. Neuroimmunol. Neuroinflamm..

[16] Sepúlveda M., Llufriu S., Martínez-Hernández E., Català M., Artola M., Hernando A., Montejo C., Pulido-Valdeolivas I., Martínez-Heras E., Guasp M. (2021). Incidence and Impact of COVID-19 in MS. Neurol. Neuroimmunol. Neuroinflamm..

[17] García L.F. (2020). Immune Response, Inflammation, and the Clinical Spectrum of COVID-19. Front. Immunol..

[18] Cajamarca-Baron J., Guavita-Navarro D., Buitrago-Bohorquez J., Gallego-Cardona L., Navas A., Cubides H., Arredondo A.M., Escobar A., Rojas-Villarraga A. (2020). SARS-CoV-2 (COVID-19) en pacientes con algún grado de inmunosupresión. Reumatol. Clin..

[19] Novi G., Mikulska M., Briano F., Toscanini F., Tazza F., Uccelli A., Inglese M. (2020). COVID-19 in a MS patient treated with ocrelizumab: Does immunosuppression have a protective role?. Mult. Scler. Relat. Disord..

[20] Kieseier B.C. (2011). The Mechanism of Action of Interferon-β in Relapsing Multiple Sclerosis. CNS Drugs.

[21] Abbadessa G., Lavorgna L., Trojsi F., Coppola C., Bonavita S. (2021). Understanding and managing the impact of the COVID-19 pandemic and lockdown on patients with multiple sclerosis. Expert Rev. Neurother..

[22] Nakhlband A., Fakhari A., Azizi H. (2021). Interferon-beta offers promising avenues to COVID-19 treatment: A systematic review and meta-analysis of clinical trial studies. Naunyn-Schmiedebergs Arch. Exp. Pathol. Pharmakol..

[23] Kalil A.C., Mehta A.K., Patterson T.F., Erdmann N., Gomez A.C., Jain M.K., Wolfe C.R., Ruiz-Palacios G.M., Kline S., Pineda J.R. (2021). Efficacy of interferon beta-1a plus remdesivir compared with remdesivir alone in hospitalised adults with COVID-19: A double-blind, randomised, placebo-controlled, phase 3 trial. Lancet Respir. Med..

[24] Tasat D.R., Yakisich J.S. (2021). Rationale for the use of sphingosine analogues in COVID-19 patients. Clin. Med..

[25] Sullivan R., Kilaru A., Hemmer B., Cree B.A.C., Greenberg B.M., Kundu U., Hach T., DeLasHeras V., Ward B.J., Berger J. (2021). COVID-19 Infection in Fingolimod- or Siponimod-Treated Patients. Neurol. Neuroimmunol. Neuroinflamm..

[26] Fan M., Qiu W., Bu B., Xu Y., Yang H., Huang D., Lau A.Y., Guo J., Zhang M.-N., Zhang X. (2020). Risk of COVID-19 infection in MS and neuromyelitis optica spectrum disorders. Neurol. Neuroimmunol. Neuroinflamm..

[27] Berger J.R., Brandstadter R., Bar-Or A. (2020). COVID-19 and MS disease-modifying therapies. Neurol. Neuroimmunol. Neuroinflamm..

[28] Louapre C., Collongues N., Stankoff B., Giannesini C., Papeix C., Bensa C., Deschamps R., Créange A., Wahab A., Pelletier J. (2020). Clinical Characteristics and Outcomes in Patients with Coronavirus Disease 2019 and Multiple Sclerosis. JAMA Neurol..

[29] Achiron A., Mandel M., Dreyer-Alster S., Harari G., Magalashvili D., Sonis P., Dolev M., Menascu S., Flechter S., Falb R. (2021). Humoral immune response to COVID-19 mRNA vaccine in patients with multiple sclerosis treated with high-efficacy disease-modifying therapies. Ther. Adv. Neurol. Disord..

[30] Capasso N., Palladino R., Montella E., Pennino F., Lanzillo R., Carotenuto A., Petracca M., Iodice R., Iovino A., Aruta F. (2020). Prevalence of SARS-CoV-2 Antibodies in Multiple Sclerosis: The Hidden Part of the Iceberg. J. Clin. Med..

[31] Safavi F., Nourbakhsh B., Azimi A.R. (2020). B-cell depleting therapies may affect susceptibility to acute respiratory illness among patients with multiple sclerosis during the early COVID-19 epidemic in Iran. Mult. Scler. Relat. Disord..

[32] Zabalza A., Cárdenas-Robledo S., Tagliani P., Arrambide G., Otero-Romero S., Carbonell-Mirabent P., Rodriguez-Barranco M., Rodríguez-Acevedo B., Vera J.L.R., Resina-Salles M. (2020). COVID-19 in multiple sclerosis patients: Susceptibility, severity risk factors and serological response. Eur. J. Neurol..

[33] Etemadifar M., Nouri H., Maracy M.R., Sigari A.A., Salari M., Blanco Y., Sepúlveda M., Zabalza A., Mahdavi S., Baratian M. (2022). Risk factors of severe COVID-19 in people with multiple sclerosis: A systematic review and meta-analysis. Rev. Neurol..

[34] Thakkar A., Gonzalez-Lugo J.D., Goradia N., Gali R., Shapiro L.C., Pradhan K., Rahman S., Kim S.Y., Ko B., Sica R.A. (2021). Seroconversion rates following COVID-19 vaccination among patients with cancer. Cancer Cell.

[35] Bar-Or A., Fawaz L., Fan B., Darlington P.J., Rieger A., Msc C.G., Calabresi P.A., Waubant E., Hauser S.L., Zhang J. (2009). Abnormal B-cell cytokine responses a trigger of T-cell-mediated disease in MS?. Ann. Neurol..

